# Applying nonlinear measures to the brain rhythms: an effective method for epilepsy diagnosis

**DOI:** 10.1186/s12911-021-01631-6

**Published:** 2021-09-24

**Authors:** Ali Torabi, Mohammad Reza Daliri

**Affiliations:** Biomedical Engineering Department, School of Electrical Engineering, Iran University of Science and Technology (IUST), 16846-13114 Narmak, Tehran, Iran

**Keywords:** Epileptic seizure, Brain rhythms, Nonlinear measures, Relieff, Neural networks, SVM

## Abstract

**Background:**

Epilepsy is a neurological disorder from which almost 50 million people have been suffering. These statistics indicate the importance of epilepsy diagnosis. Electroencephalogram (EEG) signals analysis is one of the most common methods for epilepsy characterization; hence, various strategies were applied to classify epileptic EEGs.

**Methods:**

In this paper, four different nonlinear features such as Fractal dimensions including Higuchi method (HFD) and Katz method (KFD), Hurst exponent, and L-Z complexity measure were extracted from EEGs and their frequency sub-bands. The features were ranked later by implementing Relieff algorithm. The ranked features were applied sequentially to three different classifiers (MLPNN, Linear SVM, and RBF SVM).

**Results:**

According to the dataset used for this study, there are five classification problems named ABCD/E, AB/CD/E, A/D/E, A/E, and D/E. In all cases, MLPNN was the most accurate classifier. Its performances for mentioned classification problems were 99.91%, 98.19%, 98.5%, 100% and 99.84%, respectively.

**Conclusion:**

The results demonstrate that KFD is the highest-ranking feature; In addition, beta and theta sub-bands are the most important frequency bands because, for all cases, the top features were KFDs extracted from beta and theta sub-bands. Moreover, high levels of accuracy have been obtained just by using these two features which reduce the complexity of the classification.

## Introduction

The human brain is a complex system and displays temporally intricate dynamics. One way to observe the brain’s activity is Electroencephalography. In fact, EEG signals are the recording of the brain’s electrical activity and are used by clinicians in the diagnosis of neurological disorders [1]. Epilepsy is one of the most prevailing neural diseases and almost fifty million people worldwide suffer from it [2]. Abnormal electrical discharges of a group of neurons in the brain are the cause of seizures. Therefore, EEG signals have valuable information about this disorder. Detecting abnormality in EEGs is a critical issue in the diagnosis process. Since visual inspection is not a proper and reliable method to detect abnormality in EEGs, various methods are presented to extract important features. Different kinds of strategies were used to analyze EEG data. The prevalent techniques are temporal and spectral analysis, and nonlinear methods [3]. Altunay et al. have applied the linear prediction error energy method to find seizures in EEGs. They have asserted that this approach can be used as an index for epileptic seizures in EEG signals [3, 4]. Ghosh-Dastidar et al. have used Principal Component Analysis (PCA) to classify epileptic EEGs. They proposed a model that could achieve high accuracy (99.3%) [3, 5]. Acharya et al. have used PCA with different classifiers. They obtained 99% classification accuracy using the Gaussian Mixture Model (GMM) classifier [3, 6]. Subasi and Gursoy have implemented PCA, Independent Component Analysis, and Linear Discriminant Analysis for EEGs classification [3, 7]. Lekshmi et al. utilized PCA with wavelet transforms for EEG signal classification [8]. Sharma et al. used the wavelet-statistical features method to detect non-convulsive seizures [9]. Ocak has presented an approach based on wavelet transform to classify epileptic seizures in EEG [3, 10]. A deep learning-based method was applied by Hussein et al. in order to detect seizures [11]. Raghu and Sriraam proposed a method based on neighborhood component analysis for the classification of focal seizures [12]. Mutlu employed Hilbert vibration decomposition for epilepsy diagnosis [13]. Yuan et al. used Diffusion Distance and Bayesian Linear Discriminate Analysis for predicting the seizures [14].

Amongst a wide spectrum of methods used for signal analysis, nonlinear dynamics based techniques are of great importance and have prominent information about brain signals due to EEGs’ nonlinearity and complexity. Kannathal et al. claim that entropy estimators can differentiate between normal and abnormal EEG data with the proper level of accuracy [3, 15]. Chua et al. [16] and Acharya et al. [17] applied Higher-Order Spectral (HOS) parameters for epilepsy detection [3]. The multi-fractal analysis was implemented for seizure detection [18]. Li et al. employed Fractal spectral analysis for epilepsy diagnosis [19]. Geng et al. extracted some nonlinear features (Correlation Dimension (CD), Hurst Exponent (HE), and Approximate Entropy (ApEn)) from healthy and epileptic EEGs. They declared that CD and HE are helpful in explicating epileptic EEG and interictal EEG [20]. Guler et al. have presented an algorithm using Lyapunov exponents for classifying EEGs [21].

In this study, nonlinear measures such as KFD, HFD, Hurst Exponent, and Lempel–Ziv complexity have been applied to the EEGs and brain rhythms. Relieff algorithm was used to select the best features and the classification was performed by three different classifiers (MLPNN, linear SVM, and RBF SVM). The main goal of this study is to achieve high levels of accuracy in the epileptic EEG data classification by using a few features. Besides, the most informative EEG rhythms and nonlinear features and also the best classifier for detecting epilepsy in EEGs have been determined.

### Data set

**Fig. 1:**
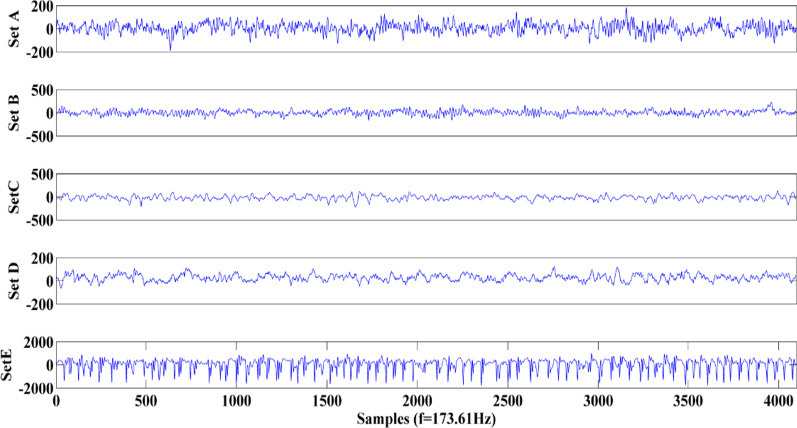
Typical example of five collections (A, B, C, D, and E). The amplitude unit for all of them is µV

The data set used for this study is publicly available online in [22] and comprises five collections signified A–E, every category consists of 100 single-channel EEG segments. The duration and sampling frequency of each segment are 23.6 s and 173.61 Hz respectively. The segments of collection A and collection B have been recorded from 5 normal subjects using 10–20 electrode system, while they were awake and relaxed with eyes open (A) and eyes closed (B). Group C consists of five patients’ recordings in seizure-free intervals from the epileptogenic zone, and group D corresponds to the hippocampal formation of the opposite hemisphere. Collection E is composed of EEG signals with seizure activity. The typical example of each EEG set is depicted in Fig. 1. More information about the data set is available in [23].

## Methods

### Classification problems

Considering these five collections, five different sub-problems including two 3-class and three binary classification problems were designed. These problems are of great practical significance and are frequently used in several research papers related to epileptic EEG classification.

1. ABCD/E
2. AB/CD/E
3. A/D/E
4. A/E
5. D/E

### Brain rhythms

**Fig. 2:**
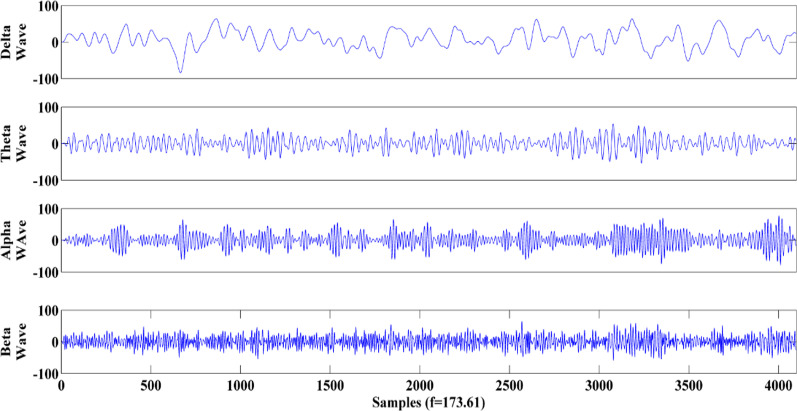
Brain rhythms for EEG data from collection A (Delta, Theta, Alpha, and Beta waves). The amplitude unit for all of them is µV

Since features will be extracted from four sub-bands, four different waves named delta rhythm (0.5–4 Hz), theta rhythm (4–8 Hz), alpha rhythm (8–14 Hz), and beta rhythm (14–30 Hz) were extracted from the original signal. Fourth-order Butterworth band-pass filters were used to extract desired frequency sub-bands. Figure 2 illustrates these waves for an EEG data sample from collection A.

### Nonlinear features

#### Fractal dimension

Fractal Dimension (FD) is a nonlinear measure which is used to analyze time series or biomedical signals. Generally, the fractal is a geometric concept referring to a set of points that has self- similarity. Fractal shapes are complex and have a non-integer dimension. FD can be calculated based on time-domain analysis or phase space domain analysis. Since phase space-based methods are very slow and time-consuming [24], we tend to apply time-domain approaches. In this paper, the methods presented by Higuchi and Katz have been reviewed and applied to EEG signals.

#### Higuchi fractal dimension (HFD)

Consider T as a temporal signal

$${\text{T}} = {\text{T}}\left( {1} \right),{\text{ T}}\left( {2} \right),{\text{ T}}\left( {3} \right), \ldots ,{\text{T}}\left( {\text{Y}} \right)$$

afterwards, p novel temporal signals are defined as follows:

1 $$\begin{gathered} T_{p}^{f} :T(f),T(f + p),T(f + 2p), \ldots ,T\left( {f + \left[ {\frac{Y - f}{p}} \right].p} \right) \hfill \\ (f = 1,2, \ldots ,p) \hfill \\ \end{gathered}$$

f shows the value of the beginning moment and p represents time intervals.

The average length is computed for all temporal signals and called A_f_ and the mean value of that (A(p)) is computed for p = 1,2,3,…, p_s_. p_s_ is the saturation point. For the current paper, two different values are considered for p_s_.

2 $${\raise0.7ex\hbox{${A_{f} (p) = \left\{ {\left( {\sum\limits_{j = 1}^{{[\frac{Y - f}{p}]}} {|T(f + jp) - T(f + (j - 1).p)|} } \right)} \right.\left. {\frac{Y - 1}{{[\frac{Y - f}{p}].p}}} \right\}}$} \!\mathord{\left/ {\vphantom {{A_{f} (p) = \left\{ {\left( {\sum\limits_{j = 1}^{{[\frac{Y - f}{p}]}} {|T(f + jp) - T(f + (j - 1).p)|} } \right)} \right.\left. {\frac{Y - 1}{{[\frac{Y - f}{p}].p}}} \right\}} p}}\right.\kern-\nulldelimiterspace} \!\lower0.7ex\hbox{$p$}}$$

3 $$A(p) = \frac{1}{p} \times \sum\limits_{f = 1}^{p} {A_{f} (p)}$$

Since

4 $$A(p) \propto p^{ - r} ,$$

by plotting log (A(p)) vs log (1/p), HFD was derived by the gradient of the straight line fitting the points [25].

#### Katz fractal dimension (KFD)

Katz proposed a method with a normalized formula for the calculation of fractal dimension. According to the Katz approach, a curve’s fractal dimension will be obtained by:

5 $$K = \frac{{\log_{10} (U)}}{{\log_{10} (b)}}$$

U is the whole length of the curve obtained by the summation of the distances between consecutive points:

6 $$U = sum(dist(j,j + 1))$$

where

7 $${\text{dist}}\left( {{\text{j}},{\text{j}} + {1}} \right) = \sqrt {(x_{j + 1} - x_{j} )^{2} + (y_{j + 1} - y_{j} )^{2} }$$

and b indicates how far apart the first point and the farthest point of the curve are:

8 $${\text{b}} = {\text{maximum}}\left( {\sqrt {(x_{1} - x_{j} )^{2} + (y_{1} - y_{\begin{subarray}{l} j \\ \end{subarray} } )^{2} } } \right)$$

Since K calculation is dependent on the units, U and b were normalized by “m” which was the mean distance betwixt successive spots. Finally, K became: [26]

9 $$K = \frac{{\log_{10} (U/m)}}{{\log_{10} (b/m)}}\mathop = \limits^{n = U/m} \frac{{\log_{10} (n)}}{{\log_{10} \left( \frac{b}{U} \right) + \log_{10} (n)}}$$

#### Hurst exponent

Hurst exponent estimates the self-similarity of a temporal signal and the values of this measure vary in the range [0, 1]; indeed, it expresses the trendiness. H > 0.5 exhibits positive correlation, while H < 0.5 displays negative correlation; H = 0.5 displays un-correlated time series [27, 28]. This feature is estimated by rescaled rang technique:

10 $$E\left[ {\frac{R(m)}{{S(m)}}} \right] = Cm^{H} \mathop {}\nolimits_{{}}^{{}} m \to \infty$$

A temporal signal of total length M is rescaled to subsequences of length m = M, M/2, M/4, M/8, …. “E” represents the expected value. R (m) and S (m) are defined as the value range and SD respectively. F is the fixed parameter. The following lines describe the rescaling:

11 $$a = \frac{1}{m}\sum\limits_{j = 1}^{m} {T_{j} }$$

12 $$W_{k} = T_{k} - a\mathop {}\nolimits_{{}}^{{}} \mathop {}\nolimits_{{}}^{{}} \mathop {}\nolimits_{{}}^{{}} k = 1,2,...,m$$

13 $$V_{k} = \sum\limits_{j = 1}^{k} {W_{j} } \mathop {}\nolimits_{{}}^{{}} \mathop {}\nolimits_{{}}^{{}} \mathop {}\nolimits_{{}}^{{}} k = 1,2,...,m$$

14 $$\frac{R}{S}(m) = \frac{R(m)}{{S(m)}} = \frac{{{\text{maximum}} (V_{1,} V_{2} ,V_{3} ,...,V_{m} ) - minimum(V_{1,} V_{2} ,V_{3} ,...,V_{m} )}}{{\sqrt {\frac{1}{m}\sum\limits_{j = 1}^{m} {(T_{j} - a)} } }}$$

After calculating R/S for all segments, they have been averaged over segments (R/S_ave_). For different values of n, different R/S_ave_ values will be obtained. The Hurst exponent can be estimated by the gradient of linear regression line wherein X and Y coordinates represent log (m) and log (R/S_ave_ (m)) respectively [29–31].

#### Lempel–Ziv (L–Z) complexity

A coarse-graining approach is the base of the L-Z complexity measure. To estimate the L-Z complexity, the temporal signal should be converted into a symbolic sequence. The binary sequence is a common choice for this purpose. This procedure is done by considering a margin for sequence values. Two different margin values (L), which were median and mean of the signal, have been used for the analysis; therefore, two different complexity values have been obtained. S is the Binary sequence of the series:

S = u(1),u(2), …,u(m).

u(j) is:

15 $$u(j) = \left\{ \begin{gathered} 0,\mathop {}\nolimits_{{}}^{{}} if_{{}}^{{}} T(j) < L \hfill \\ 1,\mathop {}\nolimits_{{}}^{{}} if_{{}}^{{}} T(j) > L \hfill \\ \end{gathered} \right.$$

The obtained binary series was scanned from left to right for both margin values. Complexity counter (c(m)) calculates the number of different substrings contained in the new sequence. As a novel string is detected, $c(m) \to c(m) + 1$.

The normalized complexity (C(m)) is defined as below [32, 33]:

16 $$C(m) = \frac{c(m)}{{m/\log_{2} (m)}}$$

### Supervised feature selection

In this part, several nonlinear features including two HFD (different k_max_s), KFD, two L-Z complexity measures (mean and median as their threshold), and Hurst exponent were extracted from not only the EEGs but also their different rhythms. Thus, the feature set has 30 members ($\{$6 nonlinear features$\}$ × $\{$5 EEGs and their rhythms$\}$). Feature selection is one of the classification steps that is of significant importance. In order to select the best subset of features, Relieff feature selection has been used. This technique selects the most significant features based on their relevance and assigns weight to features for ranking them according to their weights. More information about this algorithm is available in [34, 35].

### Classification

Three classifiers were used to perform classification on each problem: MLPNN, Linear SVM, and RBF SVM. Parameter tuning plays a major role in classifier accuracy. The classification was performed by Nested ten-fold cross-validation; one cross-validation loop for parameter setting and another loop for model selection.

#### Multi-layer perceptron neural networks (MLPNN)

An MLPNN is composed of multi-layers of computational nodes in a digraph, each layer fully connected to the next one. All nodes are considered as a neuron with an activation function except for the input nodes [36, 37]. Matlab software (R2016a) Neural Network toolbox was used for MLPNN classification. The number of neurons in the hidden layer was set by ten-fold cross-validation. The transfer function used in neural network architecture was hyperbolic tangent sigmoid.

#### Support vector machine (SVM)

Vapnik invented the SVM based on the principle of structural risk minimization. It is known as one of the most robust methods amongst the famous classification algorithms. SVM has been frequently used in various analyses such as regression, classification, and nonlinear function approximation. SVM is a binary classifier basically, but it was extended for multiclass problems by some methods [38]. We have used LIBSVM(version 3.20) tools to perform linear SVM and RBF SVM classifications [39, 40]. For more details on the SVM algorithms, please refer to papers [38, 39].

## Results and discussion

The best feature is KFD due to its rank and repetition in the selected features. The order of appearance of features for the five top features is shown in Table 1.

**Table1:** Five top rank features for each classification problems

Sub-sets	Rank				
	1	2	3	4	5
ABCD/E	KFD (Theta)	KFD (Beta)	KFD (Delta)	KFD (Alpha)	HFD _kmax=45_ (Beta)
AB/CD/E	KFD (Theta)	KFD (Beta)	HFD _kmax=30_ (Original Signal)	HFD _kmax=45_ (Original Signal)	KFD (Delta)
A/D/E	KFD (Beta)	KFD (Theta)	HFD _kmax=30_ (Original Signal)	HFD _kmax=45_ (Original Signal)	KFD (Alpha)
A/E	KFD (Theta)	KFD (Beta)	KFD (Alpha)	KFD (Delta)	KFD (Original Signal)
D/E	KFD (Beta)	KFD (Theta)	KFD (Alpha)	Hurst Exponent (Beta)	KFD (Delta)

In Table 2, performances for three different classifiers, in both selected features and all features (without feature selection), have been compared. MLPNN has the best performance in all cases. In addition, the optimal number of features for the best classification accuracy was reported.

**Table 2:** Performance for each classifier in all classification problems with and without feature selection

Classifiers	Sub-Sets					
	Linear SVM		RBF SVM		MLPNN	
	Performance with Feature Selection (No. of features)	Performance without Feature Selection (%)	Performance with Feature Selection (No. of features)	Performance without Feature Selection (%)	Performance with Feature Selection (No. of features)	Performance without Feature Selection (%)
ABCD/E	98.21% (27)	98.09	99.2% (23)	99.05	99.91% (22)	99.69
AB/CD/E	94.54% (25)	94.41	96.65% (20)	96.02	98.19% (26)	96.86
A/D/E	94.98% (19)	94.85	96.67% (18)	95.96	98.5% (27)	98.31
A/E	100% (6)	99.97	100% (5)	100	100% (5)	99.5
D/E	97.56% (26)	97.34	98.41% (19)	98.25	99.84% (23)	99.66

**Fig. 3:**
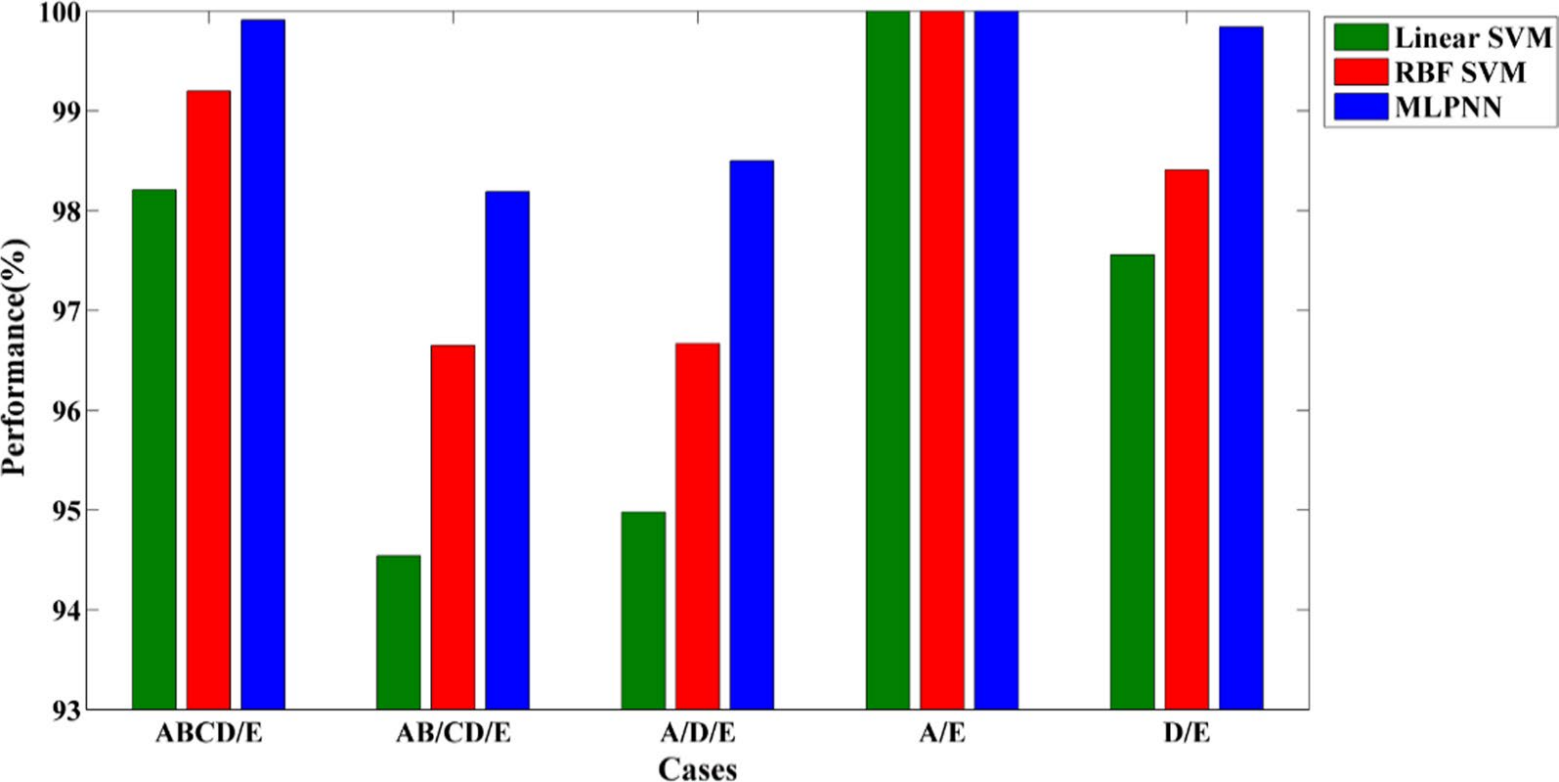
The highest performance of different classifiers (MLPNN, RBF SVM, and Linear SVM) for all classification problems. MLPNN classifier has the best classification accuracy in all cases

**Fig. 4:**
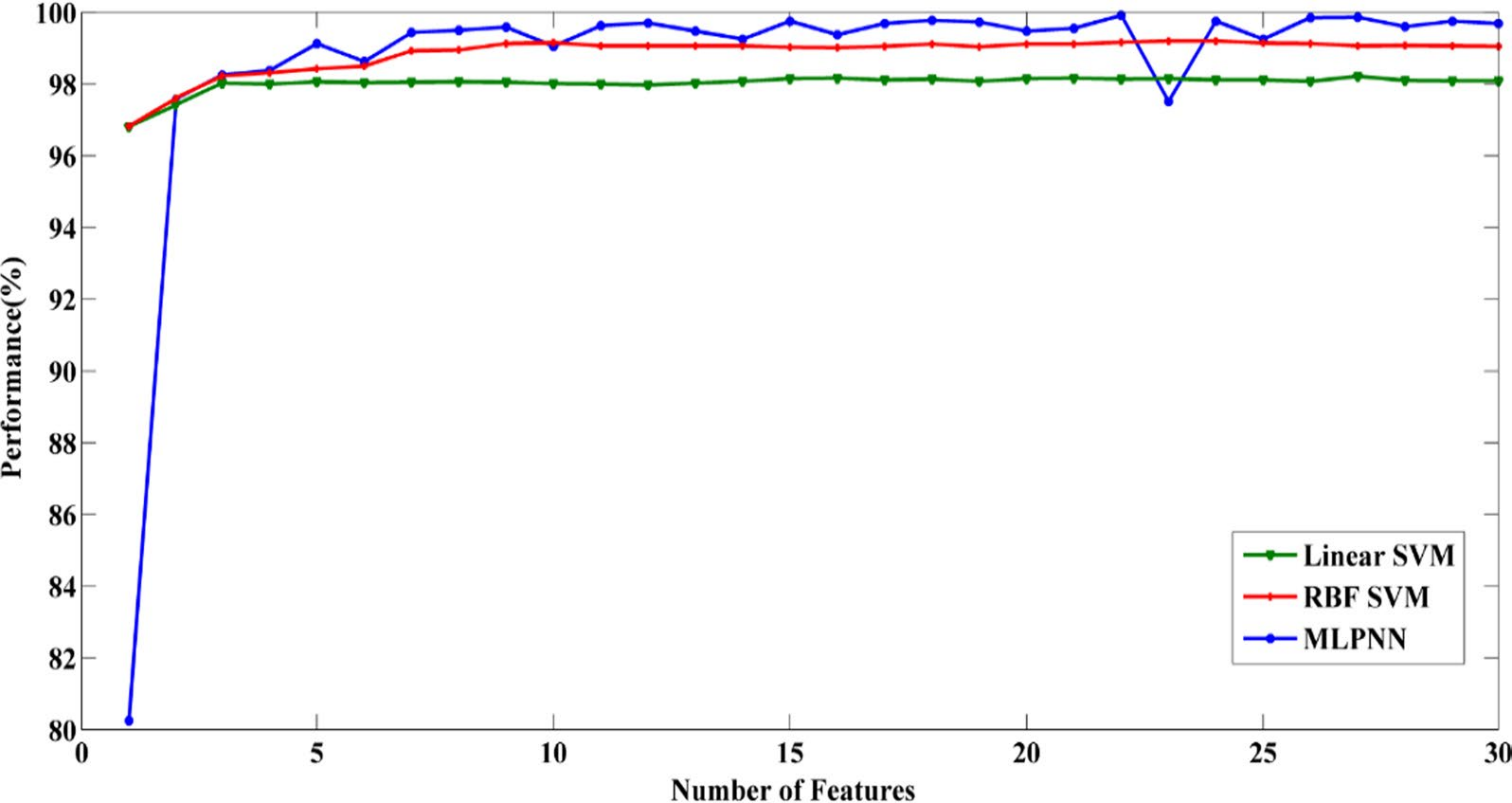
Performance versus Number of features for case1 (ABCD/E). Ranked features were applied subsequently to 3 different classifiers (MLPNN, Linear SVM, and RBF SVM). The best accuracy was obtained by the MLPNN classifier

The highest performances for three classifiers in each case are represented in Fig. 3. In Fig. 4, three classifiers are compared for case 1(ABCD / E). Although MLPNN had the best performance, when just the first selected feature was used, the accuracy of this classifier was significantly lower than other classifiers. However, when the second top feature was added to the first one, the performance was raised significantly, and finally, MLPNN reached 99.91% classification accuracy.

**Fig. 5:**
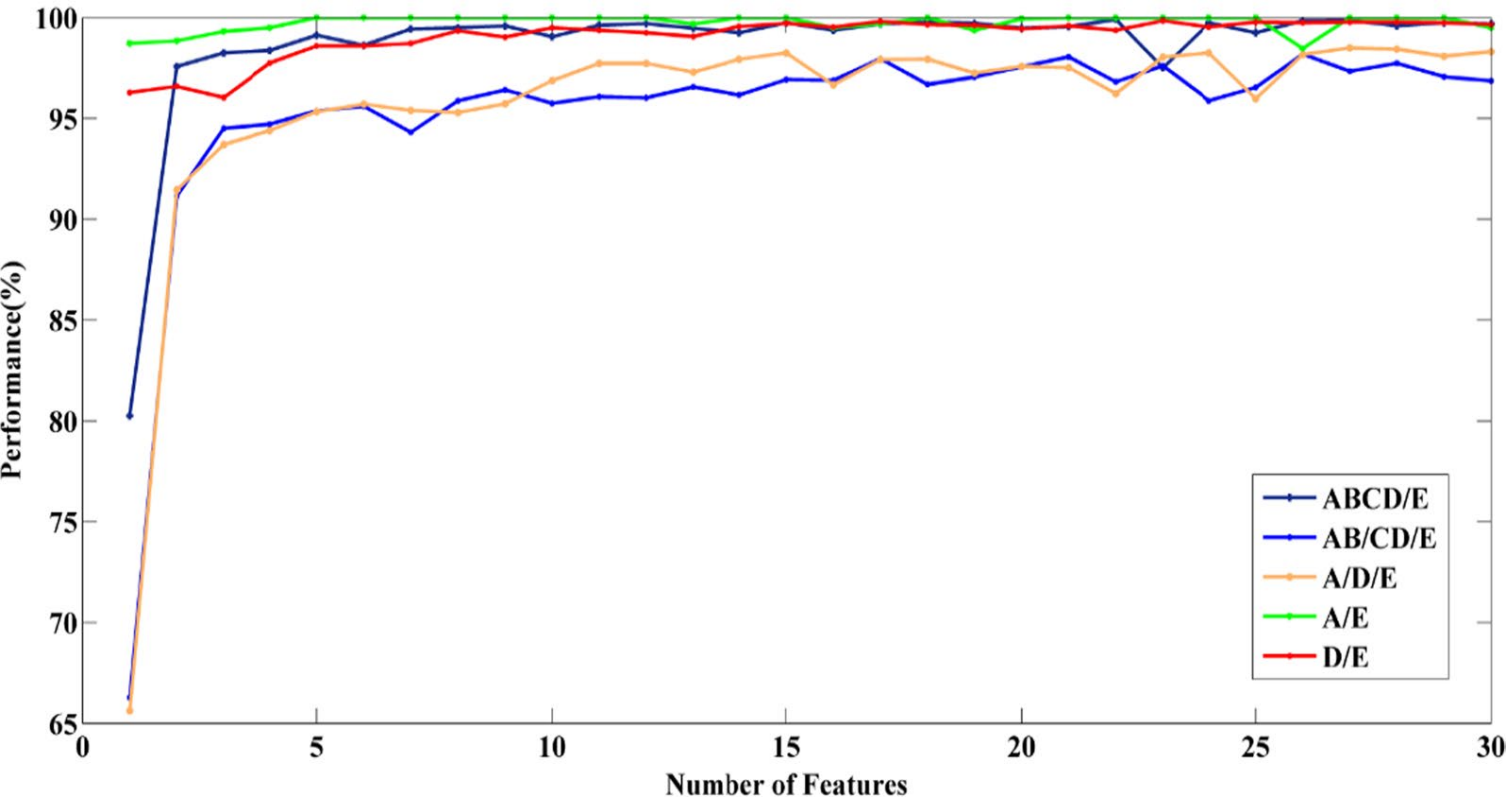
Performance versus Number of features for all cases with MLPNN classifier

Performances for MLPNN classifier are depicted in Fig. 5 for all cases. As Fig. 5 illustrates, case4 (A/E) had the best results because it could achieve the best accuracy (100%) with the lowest number of features (Optimal point (five features, 100%)). In other cases, a decent performance can be obtained by using only a few features.

Our results indicate that nonlinear features can categorize different classification problems with high accuracy and KFD is a paramount measure to classify EEGs. This shows the supremacy of the fractal dimension (computed by using the Katz algorithm). Furthermore, analysis of brain rhythms demonstrates that theta and beta frequency bands are the most informative sub-bands. By combining KFD and these two frequency bands, in other words, KFDs extracted from theta and beta rhythms, a decent level of accuracy will be achieved (just by using two features). In this study, we have used different classifiers including MLPNN, Linear SVM, and RBF SVM in order to compare the performance of them. The results reveal that the MLPNN classifier has the best classification accuracy in all of the problems. As a comparison, some of the recent studies, which were done on the same data set and the same problems, and their results are mentioned in Table3.

**Table 3:** Accuracy of some relevant studies which have used the Bonn EEG dataset. Five classification problems were mentioned for comparison

Authors	Method	Classifier	Accuracy (%)
**AB/CD/E**			
Orhan et al.[41]	Wavelet (DWT) and probability distributions by K-means clustering	ANN	95.60
Acharya et al.[42]	Entropy Measures	Fuzzy Classifier	98.1
Murugavel and Ramakrishnan[43]	Wavelet + LLE + ApEn	Hierarchical SVM	95
This work	Nonlinear features	MLPNN	98.19
**A/D/E**			
Acharya et al. [44]	Recurrence quantification analysis	SVM	95.6
Orhan et al. [41]	Wavelet (DWT) and probability distributions by K-means clustering	ANN	96.67
Wang et al. [45]	Multi-scale blanket dimension and fractal intercepts	SVM	97.13
Murugavel and Ramakrishnan [43]	Wavelet + LLE + ApEn	Hierarchical SVM	96
This work	Nonlinear features	MLPNN	98.5
**ABCD/E**			
Guo et al. [46]	Wavelet (DWT) + line length	ANN	97.7
Murugavel and Ramakrishnan [43]	Wavelet + LLE + ApEn	Hierarchical SVM	99
Orhan et al. [41]	Wavelet (DWT) and probability distributions by K-means clustering	ANN	99.60
Kumar et al. [47]	Wavelet(DWT) and Approximate Entropy	ANN, SVM	94
This work	Nonlinear features	MLPNN	99.91
**A/E**			
Guo et al. [46]	Wavelet (DWT) + line length	ANN	99.6
Orhan et al. [41]	Wavelet (DWT) and probability distributions by K-means clustering	ANN	100
Nicolaou et al. [48]	Permutation Entropy	SVM	93.55
Kumar et al. [47]	Wavelet(DWT) and Approximate Entropy	ANN, SVM	100
Wang et al. [45]	Multi-scale blanket dimension and fractal intercepts	SVM	99.83
Kaya et al. [49]	1D- local binary pattern	BayesNet, Functional Tree	99.50
Murugavel and Ramakrishnan [43]	Wavelet + LLE + ApEn	Hierarchical SVM	99
This work	Nonlinear features	MLPNN	100
**D/E**			
Kumar et al. [47]	Wavelet(DWT) and Approximate Entropy	ANN, SVM	95
Nicolaou et al. [48]	Permutation Entropy	SVM	83.13
Kaya et al. [49]	1D-local binary pattern	BayesNet, Functional Tree	95.50
This work	Nonlinear features	MLPNN	99.84

As Table3 illustrates, the binary classification problems had better performances than the three-class problems. The approach presented in this article has better performance than the other studies. Furthermore, our approach is almost homogeneous and the accuracy didn’t vary significantly by changing from the two-class problems to the three-class problems.

## Conclusion

There is some substantial information not only in seizure activity intervals of the patients’ EEGs but also in the seizure-free periods. This disorder can’t be diagnosed properly just by visual detection or simple measures. Therefore, various methods were used to classify normal and abnormal EEGs. In this study, five different classification problems were designed to classify Epileptic EEGs. These binary or three-class classification problems were used frequently in many relevant papers. We used nonlinear measures, including HFD, KFD, Hurst exponent, and L-Z complexity measure, which were applied to the original EEGs and their frequency sub-bands; then, the extracted features were ranked by Relieff algorithm. KFDs extracted from beta and theta sub-bands were the most informative features for all cases. The ranked features were applied to three different classifiers (MLPNN, Linear SVM, and RBF SVM) sequentially. Afterward, the optimum point, which had the highest accuracy with the least number of features, was detected in each problem for all classifiers. This study demonstrates that MLPNN had a better performance than the SVM classifiers; besides, the feature selection improved the accuracy of classification. The most significant advantage of this paper is that the high performances for all problems (binary and three-class cases) could be obtained just by using two features; hence, we think the proposed approach can be effectively implemented for epilepsy diagnosis thanks to its high level of accuracy with less complexity resulting of the low number of features.

## Data Availability

The data set used for this study is publicly available online in [22].
